# Unveiling Lactylation: A Novel Frontier in Cancer Stemness and Therapy

**DOI:** 10.1002/advs.202521985

**Published:** 2026-07-23

**Authors:** Ting Li, Hongyu Gu, Chang Liu, Yuesheng Lv, Feifei Li, Liang Liang, Jia Yi, Xuanyi Wang, Yi Zhun Zhu, Chuan Xu

**Affiliations:** ^1^ School of Pharmacy Faculty of Medicine Macau University of Science and Technology Macau SAR China; ^2^ Department of Oncology & Cancer Institute Sichuan Academy of Medical Sciences Sichuan Provincial People's Hospital University of Electronic Science and Technology of China Chengdu China; ^3^ Yu‐Yue Pathology Scientific Research Center Chongqing China; ^4^ Jinfeng Laboratory Chongqing China; ^5^ Collaborative Innovation Centre of Regenerative Medicine and Medical Bioresource Development and Application Co‐Constructed By the Province and Ministry Guangxi Medical University Nanning China

**Keywords:** cancer stemness, lactylation, lactylation regulatory machinery, natural products, therapeutic targeting

## Abstract

Cancer stemness represents a dynamic cellular state endowed with tumor‐initiating capacity, self‐renewal potential, and differentiation plasticity, functioning as a key driver of tumor recurrence, metastasis, and therapy resistance. Tumor metabolism and epigenetic regulation are two fundamental mechanisms that modulate cancer stemness. Lactylation, a novel post‐translational modification, has emerged as a pivotal bridge linking these two processes. Accumulating evidence highlights elevated lactylation as a critical driver of cancer stemness. To elucidate the underlying mechanisms, we systematically evaluate how lactylation orchestrates the cancer stem cell (CSC) landscape across four functional dimensions: self‐renewal maintenance, stemness‐associated signaling activation, microenvironmental adaptation, and lineage plasticity induction. Furthermore, we summarize the dynamic regulatory network governing this modification, comprising both key enzymes and indirect modulators. Beyond highlighting promising therapeutic targets and preclinical successes, we critically assess the significant challenges hindering clinical translation. This framework provides a roadmap for targeting the metabolic‐epigenetic circuitry of CSCs to combat therapy resistance and metastasis.

## Graphical abstract

Tumor metabolism and epigenetic regulation are intrinsically linked. This review highlights lactylation as a pivotal bridge connecting these processes, acting as an active epigenetic effector in cancer stemness. We delineate how lactylation orchestrates the cancer stem cell landscape and evaluate its regulatory networks. Finally, we discuss emerging pharmacological strategies and translational challenges to target lactylation.

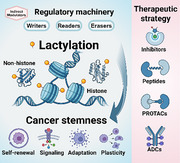

**Abbreviations**
ADCs: antibody‐drug conjugates
CSCs: cancer stem cells
ESCs: embryonic stem cells
GSCs: glioma stem cells
GTPSCS: GTP‐specific succinyl‐CoA synthetase
HDAC: histone deacetylase
KATs: lysine acetyltransferases
K_ce_
: N‐ε‐(carboxyethyl)‐lysine
K_D‐la_
: D‐lactyl‐lysine
Kla: lysine lactylation
K_L‐la_
: L‐lactyl‐lysine
lncRNAs: Long non‐coding RNAs
PDAC: pancreatic ductal adenocarcinoma
PROTACs: proteolysis‐targeting chimeras
PTMs: post‐translational modifications
TMMs: telomere maintenance mechanisms

## 1 Introduction

Cancer stemness refers to a stem‐like cellular state characterized by tumor‐initiating capacity, self‐renewal potential, and differentiation plasticity. Tumor cells possessing these traits are termed cancer stem cells (CSCs) or tumor‐initiating cells [1, 2]. Due to these attributes, CSCs can activate survival signaling pathways under therapeutic stress, allowing them to evolve into drug‐resistant populations. These treatment‐resistant cells can also enter a dormant state and later reactivate, driving tumor recurrence through self‐renewal and differentiation. Additionally, CSCs exhibit significantly higher metastatic potential than differentiated cancer cells. Collectively, these functional characteristics establish CSCs as key drivers of therapy resistance, tumor relapse, and metastasis [3].

Therefore, elucidating the mechanisms that confer such adaptability and resilience upon CSCs has become a critical scientific pursuit. Growing evidence points to metabolic plasticity as a central driver of these capabilities [4]. This plasticity allows CSCs to dynamically rewire their metabolic networks, explaining the divergent metabolic dependencies observed across different studies. These metabolic profiles range from a reliance on oxidative phosphorylation [5, 6, 7] to glycolysis [8, 9, 10]. Such flexibility enables CSCs to tailor energy production, ensuring survival amid dynamic microenvironmental and nutritional demands.

This metabolic plasticity directs research focus to its key effector molecule—lactate. Previously considered a terminal metabolic waste product of glycolysis, lactate is now recognized as a vital signaling metabolite that bridges metabolic flux and epigenetic regulation [11]. For example, it promotes tumor cell dedifferentiation and reinforces stem‐like states by enhancing histone acetylation at the Myc locus [12].

Beyond indirectly modulating such traditional marks, lactate serves as a precursor for a novel form of direct epigenetic modification: lysine lactylation (Kla). Initially identified as a histone mark induced by lactate or hypoxia, Kla regulates gene transcription and expression in macrophages under pathological conditions such as infection and cancer [13]. Subsequent studies revealed that lactylation also targets a broad range of non‐histone proteins, regulating various cancer‐related biological processes [14].

In this review, we systematically evaluate how lactylation orchestrates the CSC landscape across four key functional dimensions: core self‐renewal maintenance, stemness‐associated signaling activation, microenvironmental adaptation, and lineage plasticity induction. Next, we outline the complex regulatory network of this modification, comprising both key enzymes and indirect modulators. Finally, we critically assess current therapeutic interventions and their translational hurdles. This work aims to provide a roadmap for developing precise interventions against lactylation‐driven stemness.

## 2 Lactylation as an Active Epigenetic Effector in Cancer Stemness

Across various cancer types, bioinformatic analyses reveal that lactylation‐related gene signatures independently predict patient prognosis and therapeutic response [15, 16]. Consistent with these clinical correlations, tumor spheroids from both established cell lines and patient samples exhibit elevated global lactylation [17, 18, 19].

However, these observations primarily establish a strong correlation between elevated lactylation and cancer stemness. A critical question has thus emerged: is lactylation a functional driver of this stem‐like state, or merely a byproduct of accelerated glycolytic flux? Emerging interventional evidence suggests that lactylation functions as an active epigenetic effector rather than a passive marker of metabolic flux.

Loss‐of‐function studies provide direct causal evidence. Genetic depletion of the lactyltransferase p300 not only reduces global protein lactylation levels but also downregulates stemness marker expression and impairs CSC survival [19, 20]. In vivo, site‐directed mutagenesis of specific lactylation sites has been shown to reduce CSC‐related growth and tumor‐initiating capacity in xenograft models [19]. Beyond these global interventions, this functional necessity is underscored by locus‐specific regulation. Histone lactylation marks are significantly enriched at the promoters of core stemness genes (*OCT4*, *CD44*, and *c‐MYC*) [19, 21]. This spatial specificity is further validated by targeted epigenetic editing. Selectively erasing lactylation at these promoters using a dCas9‐HDAC3 fusion system leads to a significant reduction in their expression, crucially without altering the global metabolic state [21].

## 3 Mechanisms of Lactylation‐Mediated Cancer Stemness

Lactylation has emerged as a pervasive modification targeting not only histones but also an expanding repertoire of non‐histone proteins, including transcription factors, metabolic enzymes, and DNA repair proteins. The regulatory impact of lactylation on cell fate is well‐established in normal stem cell models. For instance, during pluripotency induction, the totipotency‐associated transcription factor Dux and the pluripotency regulator Glis1 facilitate H3K18la enrichment at the promoters of core pluripotency genes (e.g., *Oct4*, *Sall4*, and *Mycn*), thereby promoting reprogramming [22, 23]. Beyond histone marks, direct lactylation of key transcription factors is crucial for cellular identity. In embryonic stem cells (ESCs), lactylation of the master regulator Esrrb enhances its DNA‐binding affinity to target promoters like *Nanog* and *Tbx3*, sustaining self‐renewal capacity [24].

These observations suggest that malignant cells may co‐opt similar metabolic‐epigenetic circuits to maintain or acquire stemness characteristics in response to metabolic shifts within the tumor microenvironment. Inspired by these conceptual parallels, a growing body of research has begun to unravel the specific roles of lactylation in CSCs. To synthesize these emerging findings, we systematically dissect the underlying mechanisms across four functional dimensions: (i) regulation of core self‐renewal networks, (ii) modulation of stemness‐associated signaling pathways, (iii) orchestration of stress responses and microenvironmental adaptation, and (iv) induction of lineage plasticity and phenotypic reprogramming.

### 3.1 Regulation of the Core Self‐Renewal Network

The hallmark of cancer stemness is the capacity for indefinite self‐renewal, a process primarily governed by a core network of transcription factors. Recent evidence highlights histone lactylation as a critical upstream regulator that reinforces this network through transcriptional activation of stemness‐associated genes. For instance, in colorectal cancer, hypoxia‐triggered H3K18la is markedly enriched at the promoters of *OCT4*, *CD44*, and *c‐MYC*, directly driving their transcription [25]. This mechanism is complemented by other histone marks; liver CSCs exhibit significantly elevated levels of H3K56la, which similarly occupies the promoter region of OCT4 to sustain its expression [19]. Furthermore, in gastric adenocarcinoma, a feedback loop driven by the deubiquitinase PSMD14 promotes lactate accumulation and subsequent H3K27la enrichment at the SOX9 promoter, an epigenetic activation critical for the self‐renewal of these cells [26].

To ensure the robustness of the self‐renewal network, lactylation extends its influence beyond direct chromatin remodeling to include the post‐translational modulation of the transcription factors themselves. For example, in non‐small cell lung cancer, hypoxia induces the direct lactylation of the transcription factor SOX9. This modification is closely associated with elevated SOX9 protein levels and subsequently drives glycolysis‐dependent cancer stemness, migration, and invasion [27]. In endometrial cancer, the lactylation of the transcription factor SP1 at the K19 residue enhances its binding affinity to the promoter region of *CENPL*. This transcriptional activation drives *CENPL* expression, which subsequently functions as a stabilizer to maintain the abundance of the core stemness factor SOX2 by preventing its degradation [28].

The functional integrity of this core network is further safeguarded by lactylation‐mediated spatial reorganization and protein stability. Lactylation can alter signaling outcomes by regulating the spatial distribution or binding partners of key proteins. In hepatocellular carcinoma (HCC), the lactylation of the glycolytic enzyme aldolase A (ALDOA) at K322 and K230 disrupts its sequestration of the RNA helicase DDX17. This release enables DDX17 to translocate into the nucleus and function as a co‐activator for SOX2 [19]. Similarly, in breast cancer, lactylation of zinc finger MIZ‐type containing 1 (ZMIZ1), a critical co‐activator of Notch signaling, at K843 prevents its own degradation by influencing its SUMOylation and ubiquitination. The resulting stabilization of ZMIZ1 enhances the transcriptional activity of NANOG, thereby promoting tamoxifen resistance and stemness [29].

Beyond nuclear proteins, lactylation also targets transmembrane receptors to regulate stemness. In colorectal cancer, lactate shuttled from cancer‐associated fibroblasts via monocarboxylate transporters induces the lactylation of anthrax toxin receptor 1 (ANTXR1) at K453. This modification enhances the stability of the ANTXR1 protein, which subsequently activates the RhoC/ROCK1/SMAD5 signaling axis. As a transcriptional regulator, SMAD5 is recruited to the promoters of the CSC markers *LGR5* and *CD44*, sustaining the stem‐like state and conferring chemoresistance [30].

### 3.2 Modulation of Stemness‐Associated Signaling Pathways

While the epigenetic activation of core transcription factors establishes the foundation for CSC self‐renewal, sustaining this state requires continuous input from oncogenic signaling networks. Beyond its direct transcriptional control, lactylation facilitates the aberrant activation of key signaling cascades. The Wnt/β‐catenin pathway is particularly susceptible to lactylation‐mediated modulation through both indirect and direct mechanisms. In lung adenocarcinoma, H3K18la upregulates the m6A reader YTHDF2, which in turn degrades *SFRP2* mRNA to relieve the inhibition on the Wnt/β‐catenin pathway, thereby promoting a stem‐like state [31, 32]. Direct modification of signaling proteins further enhances this pathway. In hypoxic colorectal cancer, elevated β‐catenin lactylation inhibits its degradation, leading to its cellular accumulation and the sustained activation of Wnt‐driven stemness programs [33].

In triple‐negative breast cancer (TNBC), the regulation of the Wnt/β‐catenin pathway is even more intricately orchestrated. The biphenyl hydrolase‐like (BPHL) protein actively recruits POLR2A (the catalytic subunit of RNA polymerase II) and physically interacts with it to inhibit its lactylation at the K1125 residue. Concurrently, BPHL recruits the E3 ubiquitin ligase BARD1 to the same site, leading to the ubiquitination and proteasomal degradation of POLR2A. The resulting reduction in POLR2A levels alleviates R‐loop accumulation, a process that subsequently triggers the nuclear translocation of β‐catenin. This axis ultimately drives the transcription of core pluripotency factors, such as *OCT4*, *SOX2*, and *c‐MYC*, to sustain the self‐renewal of TNBC stem cells [34].

Beyond the Wnt cascade, lactylation serves as a metabolic sensor for glycolytic flux, facilitating the transcription of a broader spectrum of signaling molecules. In pancreatic ductal adenocarcinoma, Sirtuin 4 (SIRT4) stimulates glycolysis by activating the enzyme ENO1. The resulting increase in lactate production elevates histone lactylation levels, particularly H3K9la and H3K18la. Integrated RNA‐seq and CUT&Tag analyses revealed that these modifications promote the transcription of diverse genes involved in stemness‐related signaling, including *PIK3CD*, *WNT2B*, *FGFR4*, *JAK3*, and *MAPK11* [35].

Collectively, these findings illustrate how lactylation functions as a metabolic‐epigenetic bridge, translating glycolytic flux into the sustained activation of oncogenic signaling networks that maintain cancer stemness.

### 3.3 Orchestration of Stress Responses and Microenvironmental Adaptation

The establishment of self‐renewal circuits and oncogenic signaling networks provides CSCs with a fundamental baseline for survival. However, tumor progression ultimately demands adaptive resilience against external therapeutic interventions and fluctuating microenvironmental stresses. In this context, lactylation functions as a critical transducer that converts metabolic shifts into survival signals, enabling CSCs to evade cell death and adapt to hostile niches.

#### 3.3.1 Fortifying Therapeutic Resistance

Lactylation significantly promotes tumor resistance to radiotherapy and chemotherapy by activating cytoprotective mechanisms. In colorectal cancer stem cells (CCSCs), H4K12la serves as a critical driver of chemoresistance. The acetyltransferase p300 and the delactylase HDAC1 coordinately regulate H4K12la enrichment at the promoter region of the glutamate‐cysteine ligase catalytic subunit (GCLC). As the rate‐limiting enzyme in glutathione synthesis, GCLC upregulation effectively suppresses ferroptosis, thereby enhancing CCSC resistance to oxaliplatin [17]. This defensive paradigm extends to other malignancies. In liver cancer, H3K18la‐mediated transcriptional activation of the DNA replication licensing factor MCM7 upregulates *OCT4* and *NANOG*, thereby fortifying the stem‐like phenotype and inducing radioresistance [36].

Similar defensive mechanisms are observed in glioma stem cells (GSCs). H3K18la facilitates the transcriptional activation of the deubiquitinase USP4, which subsequently stabilizes and activates the ANXA2 protein. This relay triggers the BMX‐STAT3 signaling axis [37], promoting the transcription of anti‐apoptotic genes (e.g., BCL‐2) [38] and core stemness factors (e.g., SOX2) [39], which ultimately enhances the self‐renewal capacity and radioresistance of GSCs.

#### 3.3.2 Maintaining Genomic Stability and Internal Homeostasis

Beyond conferring defense against external treatments, lactylation safeguards the long‐term survival of CSCs by maintaining genomic integrity and modulating internal homeostasis. In liver CSCs, hepatitis B virus (HBV)‐induced activation of hepatic stellate cells promotes multi‐site lactylation of PARP1. This modification reinforces telomere maintenance mechanisms (TMMs), ensuring genomic stability and the immortalized characteristics of the CSC population [40].

Furthermore, lactylation enables CSCs to fine‐tune their internal stress responses. In endometrial cancer, the hypoxia‐induced H3K18la‐NHE7‐COX6C axis promotes oxidative phosphorylation to drive adaptive endoplasmic reticulum (ER) stress, which sustains cellular survival and fosters malignant progression [41]. This adaptive resilience is often reinforced by self‐sustaining loops. For instance, H3K18la upregulates the long non‐coding RNA *LINC01127*, which directly recruits POLR2A to the *MAP4K4* promoter to activate the JNK pathway. This signaling cascade leads to IκBα phosphorylation and NF‐κB reactivation, further stimulating lactate production and sustaining a persistent metabolic‐epigenetic feedback circuit [42].

A parallel self‐reinforcing mechanism is evident in GSCs through the modification of polypyrimidine tract‐binding protein 1 (PTBP1). Lactylation of PTBP1 at K436 attenuates its interaction with the E3 ubiquitin ligase TRIM21, preventing ubiquitin‐mediated degradation and sustaining PTBP1 protein levels. Concurrently, this modification strengthens the binding affinity of PTBP1 to the 3’UTR of *PFKFB4* mRNA, enhancing its stability. As a key glycolytic enzyme, the resulting overexpression of PFKFB4 creates a positive feedback loop that intensifies both glycolytic flux and the stemness phenotype of GSCs [43].

#### 3.3.3 Paracrine Remodeling of the Tumor Niche

While intracellular adaptive mechanisms ensure individual CSC survival, lactylation extends its influence outward, facilitating the remodeling of the tumor microenvironmental niche through paracrine signaling. PDP1 upregulation prevents H3K18la‐mediated activation of *DNMT1*, thereby protecting *ADCY5* from methylation‐associated silencing. The sustained expression of ADCY5 triggers cAMP/Ca^2^^+^ signaling and the senescence‐associated secretory phenotype (SASP). These paracrine factors remodel the local niche and induce epithelial‐mesenchymal transition (EMT) in neighboring cells, ultimately reinforcing the stemness landscape at a collective level [44].

### 3.4 Induction of Lineage Plasticity and Phenotypic Reprogramming

Beyond remodeling the local niche through paracrine signaling, the dynamic nature of CSCs extends to intracellular phenotypic switching. In this context, lactylation emerges as a key driver of lineage plasticity, enabling cancer cells to undergo state transitions in response to microenvironmental pressures.

This role is particularly prominent in adenocarcinoma‐to‐neuroendocrine transdifferentiation (NED). Research has demonstrated that the Numb/Parkin axis maintains mitochondrial fitness and constrains glycolytic flux through mitophagy; its deficiency triggers a “metabolic‐epigenetic switch” driven by lactate accumulation and elevated histone lactylation. This modification directly activates the transcription of core neuroendocrine genes, including *ASCL1*, *CHGA*, and *SYP*, thereby driving the transition of prostate and lung adenocarcinoma cells toward a neuroendocrine phenotype [45].

These epigenetic switches also complement specific transcription‐factor‐driven pathways. In the context of ZEB1‐induced metabolic reprogramming in prostate cancer, lactate accumulation enhances H3K18la modifications at the loci of the pluripotency factor *Myc* and neuroendocrine markers *Chga* and *Eno2* [46]. Collectively, lactylation acts as a direct signal for cell fate determination. It translates metabolic changes into stable transdifferentiation programs, ultimately driving phenotypic evolution and therapeutic evasion.

**FIGURE 1:**
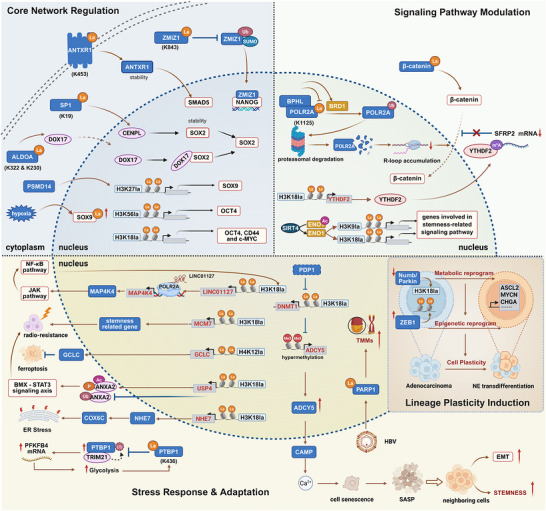
Multidimensional mechanisms of lactylation in orchestrating the cancer stem cell phenotype. Instead of being a passive consequence of lactate accumulation, lactylation functions as an active regulatory relay that translates glycolytic signals into the cohesive orchestration of the cancer stem cell (CSC) phenotype. This schematic highlights how lactylation drives cancer stemness across four functional dimensions: 1) **Core Network Regulation**: Lactylation utilizes diverse mechanisms to functionally converge on the core stemness machinery. This encompasses the direct epigenetic activation of genes like OCT4 and SOX9 via histone marks, the post‐translational modification of non‐histone factors (e.g., SOX9 and SP1), and the modulation of protein stability or sequestration (e.g., ZMIZ1, ANTXR1, and the ALDOA/DDX17 relay) to ensure the robust and persistent activity of the core self‐renewal network. 2) **Signaling Pathway Modulation**: Lactylation facilitates the persistent activation of key oncogenic signaling cascades. The Wnt/β‐catenin pathway is sustained through the direct lactylation of β‐catenin, as well as through indirect epigenetic networks involving H3K18la‐mediated YTHDF2 upregulation and BPHL‐regulated POLR2A degradation. Furthermore, SIRT4‐driven glycolysis elevates histone lactylation levels to broadly activate downstream signaling components involved in stemness. 3) **Stress Response and Adaptation**: Lactylation acts as a critical transducer enabling CSCs to adapt to hostile niches and therapeutic interventions. It fortifies defense mechanisms against ferroptosis (via H4K12la and GCLC) and radiotherapy (via H3K18la, MCM7, and the USP4/ANXA2/BMX‐STAT3 relay). Additionally, lactylation drives adaptive ER stress, maintains genomic stability through PARP1, and remodels the tumor niche via paracrine signaling and the senescence‐associated secretory phenotype (SASP). 4) **Lineage Plasticity Induction**: Functioning as a core instructive signal for cell fate reprogramming, elevated histone lactylation—resulting from metabolic reprogramming induced by ZEB1 or Numb/Parkin deficiency—directly activates the transcription of core neuroendocrine genes (e.g., ASCL2, MYCN, and CHGA). This reprogramming drives the lineage transition from adenocarcinoma to neuroendocrine (NE) transdifferentiation.

In summary, the evidence presented in this section highlights lactylation as a key link between elevated glycolysis and the maintenance of cancer stemness. By driving the transcriptional activation and functional dynamics of core stemness factors, sustaining oncogenic signaling, orchestrating adaptive resilience and microenvironmental remodeling, and instructing lineage plasticity, lactylation proves essential for the long‐term survival and adaptability of CSCs (Figure 1).

Despite these advances, the increasing complexity of these networks presents a new conceptual bottleneck: defining the precise regulatory hierarchy of lactylation. Current evidence points to a highly context‐dependent function. While precision interventions such as epigenetic editing highlight lactylation as a rate‐limiting switch at specific loci, it frequently acts as a cooperative layer integrated within a broader, multi‐dimensional regulatory landscape. Consequently, a central challenge for future research is distinguishing whether lactylation initiates stemness programs as a primary driver or sustains them as a secondary metabolic reinforcement.

To resolve this hierarchical ambiguity, the field must transition from relying on global metabolic perturbations and broad‐spectrum inhibitors toward the deployment of high‐resolution genetic and locus‐specific epigenetic editing tools. Dissecting the exact functional weight of lactylation within these complex circuits is not merely a technical necessity, but a crucial step for advancing from descriptive mechanistic studies to definitive causal models in CSC biology.

### 3.5 The Uniqueness of Lactylation in the Metabolic PTM Landscape

Metabolic reprogramming endows cancer stem cells (CSCs) with the plasticity to rewire their bioenergetic pathways, generating a diverse pool of reactive intermediates—including acetyl‐CoA, succinyl‐CoA, crotonyl‐CoA, and lactate—which serve as direct precursors for post‐translational modifications (PTMs). These metabolic‐epigenetic PTMs act as dynamic sensors that translate metabolic states into enduring gene expression programs. A synthesis of current evidence reveals that lactylation exhibits distinct substrate‐level, functional, and regulatory characteristics compared to other metabolism‐derived PTMs in CSC regulation.

At the substrate level, lactylation benefits from direct precursor utilization and a substantial concentration advantage. Modifications such as acetylation, succinylation, and crotonylation rely on acyl‐CoA precursors. In contrast, recent findings indicate that certain lactyltransferases, such as AARS1, can utilize lactate directly as a substrate [47]. Furthermore, the intracellular availability of these substrates differs by several orders of magnitude. Acyl‐CoA pools typically fluctuate in the micromolar or nanomolar range in response to transient energy demands. Conversely, in the hypoxic tumor niche, intracellular lactate accumulates to levels of 10–30 mm [48, 49, 50]. This persistent accumulation provides a stable substrate supply, overcoming the rapid turnover characteristic of acyl‐CoAs. Consequently, lactylation serves as an efficient epigenetic relay that CSCs can utilize to sustain stemness programs under metabolic stress.

Functionally, the impact of acetylation on CSCs is highly context‐dependent, whereas current evidence suggests that lactylation serves as a consistent positive regulator. For example, while acetyl‐CoA accumulation can promote stemness by activating the MYC oncogene [12], specific acetylation events also exert inhibitory effects. Gut microbiota‐derived butyrate, acting as an HDAC inhibitor, elevates H3K9ac levels to transcriptionally activate *ZFP36*; this accelerates *LRP5* mRNA decay and blocks the Wnt/β‐catenin pathway, thereby dampening breast cancer stemness [51]. In contrast to the dual nature of acetylation, lactylation is predominantly linked to the sustained activation of core stemness programs across various cancer types.

Within the context of CSC maintenance, current investigations into succinylation and crotonylation primarily focus on their roles in localized adaptive responses. For instance, these modifications facilitate CSC survival by modulating lipid desaturation [52] or fatty acid oxidation [53] to maintain intracellular homeostasis. Additionally, histone crotonylation supports survival by repressing immunogenic transposable elements to evade immune surveillance [54]. In contrast, rather than merely supporting these localized metabolic and immunological baselines, lactylation extends its influence to actively drive global microenvironmental remodeling, lineage plasticity, and the activation of core stemness programs. Consequently, lactylation demonstrates a broader regulatory scope than succinylation and crotonylation.

Moreover, lactylation is distinguished by the presence of specialized, self‐sustaining positive feedback loops. It directly activates specific glycolytic enzymes (e.g., PFKFB4) and signaling scaffolds (e.g., LINC01127) [42, 43]. These targets explicitly amplify further lactate production, creating a continuous feed‐forward cycle where lactate accumulation fuels lactylation, which in turn drives more lactate generation. Consequently, this feed‐forward loop provides a highly efficient mechanism for CSCs to maintain the stem‐like phenotype.

Adding to this regulatory depth, lactylation exhibits a unique structural complexity, existing as three distinct stereochemical isomers: L‐lactyl‐lysine (K_L‐la_), D‐lactyl‐lysine (K_D‐la_), and N‐ε‐(carboxyethyl)‐lysine (K_ce_) [55]. Although the isomer‐specific functions in cancer stemness remain to be fully elucidated, this structural diversity provides a potential molecular basis for the high‐resolution, specific regulation of the metabolism‐epigenetics‐stemness axis, further distinguishing it from simpler acylations.

## 4 Therapeutic Targeting of Lactylation

Targeting CSCs is regarded as a cornerstone for overcoming therapy resistance and preventing tumor relapse; however, current therapeutic modalities for effectively depleting these cells remain limited. Major challenges include the lack of specific CSC surface markers and the overlapping roles of core stemness‐maintaining signaling pathways in normal adult stem cell physiology. Consequently, therapies directed against these shared networks frequently disrupt normal tissue homeostasis, resulting in narrow therapeutic windows.

Emerging evidence reveals that CSCs exhibit markedly upregulated lactylation levels compared to differentiated tumor cells. While this accumulation suggests a potential therapeutic opportunity, the clinical translation of targeting lactylation faces formidable challenges. Most known lactylation regulators, such as p300 and various HDACs, serve as broad‐spectrum modifiers with essential roles in normal stem cell niches [56] and immune surveillance—notably functioning as critical epigenetic regulators for T cell activation [57]. Indiscriminate inhibition of these enzymes therefore poses a severe risk of systemic toxicity and immune suppression. Furthermore, a critical translational gap persists between identifying mechanistic vulnerabilities and developing druggable, site‐specific therapeutic agents.

**FIGURE 2:**
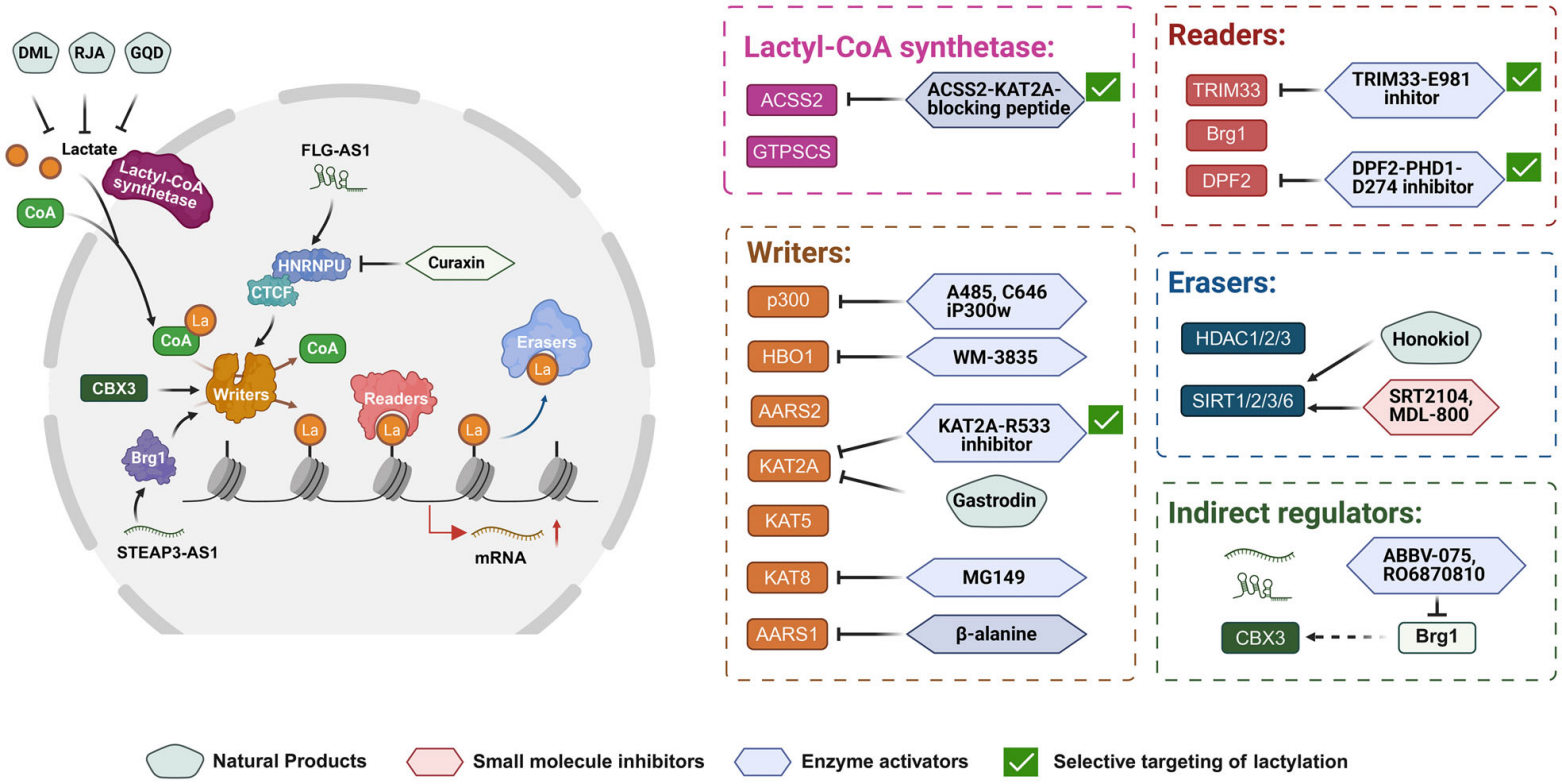
Integrated Regulatory Network and Pharmacological Landscape of Lactylation. This figure systematically illustrates the dynamic regulatory axis of lactylation, ranging from metabolic substrate supply and molecular recognition to multi‐level pharmacological interventions: 1) **Substrate Pathways and Metabolic‐Epigenetic Coupling**: cytoplasmic lactate is converted into the substrate lactyl‐CoA by ACSS2 or GTPSCS, which is subsequently utilized by the KAT2A‐ACSS2 complex to catalyze histone lactylation in the nucleus. Additionally, AARS1 functions as an intracellular lactate sensor to mediate protein lactylation, a process that can be competitively inhibited by β‐alanine. 2) **Core Regulatory Machinery and Spatial Regulation**: lactylation levels are dynamically governed by “writers” (p300, KAT2A, MYST family), “readers” (DPF2, TRIM33, Brg1), and “erasers” (HDACs, SIRTs). lncRNAs (e.g., STEAP3‐AS1) and the molecular adapter CBX3 synergistically facilitate the precise spatial recruitment of the core machinery to specific genomic loci. 3) **Pharmacological Targeting Strategies**: (1) Broad‐spectrum and Mechanistic Modulation: inhibitors targeting the p300/MYST families (e.g., A‐485, MG149) and delactylase activators (e.g., SRT2104, MDL‐800). (2) Site‐ or Mechanism‐Specific Interventions: development of site‐specific strategies exploiting unique structural recognition pockets, such as inhibitory approaches targeting KAT2A‐R533, DPF2‐D274, or TRIM33‐E981, as well as the ACSS2‐KAT2A blocking peptide designed to decouple specific metabolic‐epigenetic interactions. 4) **Regulatory Roles of Natural Products**: *Royal jelly acid* (RJA), *Demethylzeylasteral* (DML), and *Ge Gen Qin Lian Decoction* (GQD) reduce lactylation levels by inhibiting the glycolytic pathway and decreasing lactate production. Furthermore, *gastrodin* modulates lactylation by inhibiting KAT2A, while *honokiol* contributes to the downregulation of this modification by activating the delactylase SIRT3.

Against this backdrop, this section critically evaluates current strategies for the therapeutic targeting of the lactylation regulatory machinery, spanning from diverse synthetic agents to putative natural compounds. Alongside highlighting preclinical successes, we delineate key mechanistic hurdles and target‐specific limitations, including protein druggability and inhibitor selectivity (Figure 2).

### 4.1 Targeting the Core Lactylation Machinery: Writers, Erasers, and Readers

Similar to other PTMs, the protein lactylation landscape is dynamically orchestrated by a tripartite biochemical framework consisting of “writers”, “readers”, and “erasers”. While these regulators provide theoretically novel nodes for disrupting the metabolic‐epigenetic circuitry of CSCs, their therapeutic exploitation remains in its infancy. Transforming these biologically significant enzymes into viable drug targets necessitates a critical evaluation of several significant challenges, including their pleiotropy, associated toxicity risks in somatic stem cells, and the translational gap between molecular recognition and clinical druggability.

#### 4.1.1 Targeting Writers: The Challenge of Selectivity

Lysine acetyltransferases (KATs), traditionally recognized as writers of histone acetylation, have recently been shown to catalyze Kla. Among the major human KAT families, p300 (also known as KAT3B) extensively catalyzes the lactylation of both histone and non‐histone proteins. This activity drives tumor progression by maintaining cancer stemness, promoting proliferation, and facilitating immune evasion [17, 58, 59]. Consequently, pan‐p300/CBP inhibitors such as A‐485, C646, and iP300w have demonstrated significant anti‐tumor efficacy and the ability to reduce intracellular lactylation levels in various preclinical models [22, 37, 60, 61, 62].

However, the clinical translation of these inhibitors is hindered by formidable challenges regarding druggability and selectivity. A primary caveat is that p300 functions as a dual‐purpose writer for both acetylation and lactylation. Current small‐molecule inhibitors like A‐485 lack the selectivity required to distinguish between these two modifications. Consequently, systemic administration risks broad‐spectrum epigenetic dysregulation across normal tissues—specifically disrupting normal hematopoietic stem cell (HSC) homeostasis and T cell activation [56, 57]. This hematological toxicity and immune suppression severely narrow the therapeutic window.

Recent breakthroughs have identified KAT2A (GCN5) as a bona fide histone lactyltransferase that catalyzes H3K14 and H3K18 lactylation [63, 64]. Unlike the broader‐spectrum p300, structural analyses reveal that KAT2A relies on the R533 residue within its catalytic pocket to specifically accommodate the lactyl moiety of lactyl‐CoA. This unique residue provides a clear structural basis for distinguishing lactylation from acetylation at the molecular level.

Beyond direct catalytic inhibition, strategies to modulate KAT2A protein stability have also emerged. For instance, the natural compound Gastrodin (GAS), a phenolic glycoside extracted from the orchid plant Gastrodia elata, has been found to enhance the interaction between KAT2A and CDT2 (Cdc10‐dependent transcript 2), thereby promoting ubiquitin‐mediated degradation of KAT2A and consequently reducing H3K14la [65]. Although these findings highlight specific regulatory nodes, selectively targeting these interfaces without impairing KAT2A's essential acetylation functions remains a formidable medicinal chemistry challenge.

Other members of the MYST family also exhibit lactyltransferase activity, but their potential systemic toxicity warrants careful consideration. For instance, HBO1 has been shown to promote the expression of genes essential for hematopoietic stem cell quiescence and self‐renewal [66]. This implies that while interventions targeting HBO1 might weaken CSC stemness, they are also highly likely to disrupt the stability of the normal hematopoietic system. Similarly, KAT5/TIP60 and KAT8/MOF have also exhibited lactyltransferase activity toward histones or specific substrates like NBS1 and eEF1A2 [67, 68]. The long‐term safety and lineage selectivity of their inhibitors (e.g., WM‐3835 and MG149) in CSC subpopulations require stringent validation.

Furthermore, members of the aminoacyl‐tRNA synthetase (ARS) family are pivotal for non‐histone lactylation [47]. Beyond AARS1‐mediated regulation of core factors like p53 (K120/K139) [69] and YAP (K90) [70], its mitochondrial homolog AARS2 catalyzes the lactylation of PDHA1 (K336) and CPT2 (K457/458), thereby inhibiting their enzymatic activity and suppressing oxidative phosphorylation [71]. To target this pathway, β‐alanine has emerged as an effective Kla inhibitor due to its structural mimicry of lactate and its ability to competitively bind AARS1 [69, 70]. Notably, physiological levels of alanine do not suppress lactylation; rather, only exogenous supplementation of β‐alanine effectively inhibits this modification through a competitive mechanism [71]. Although β‐alanine supplements have a proven safety profile in humans [72], their clinical application requires rigorous pharmacokinetic evaluation to determine the therapeutic index and ensure they do not interfere with broader amino acid metabolism or normal protein translation.

#### 4.1.2 Targeting Erasers: The Risk of Epigenetic Perturbation

The removal of Kla marks is dynamically orchestrated by “erasers” primarily belonging to the histone deacetylase (HDAC) superfamily, which includes zinc‐dependent HDACs and NAD^+^‐dependent sirtuins (SIRTs). Among the zinc‐dependent family, HDAC1, HDAC2, and HDAC3 have been identified as functional delactylases [73], though they exhibit distinct functional heterogeneity across different tumor types and microenvironments. For instance, while HDAC3 effectively removes H3K18la modifications in colorectal and breast cancer models [74, 75], its overexpression in pancreatic ductal adenocarcinoma (PDAC) cells does not significantly alter global or H3K18la levels. In the context of PDAC, HDAC2 emerges as the dominant delactylase instead [76]. Further studies indicate that HDAC2‐mediated suppression of H3K9 lactylation not only regulates tumor cell behavior but also inhibits the angiogenic capacity of endothelial cells [77].

Within the SIRT family, SIRT1–3 and SIRT6 have been characterized as potent delactylases [78, 79, 80]. Notably, SIRT1 has been shown to remove lactylation marks from both histone H3K18 and the non‐histone protein PTBP1. Its small‐molecule activator, SRT2104, stabilizes the SIRT1 protein, thereby inhibiting gastric cancer cell growth and impairing the stemness maintenance of GSCs [43, 81]. Furthermore, SIRT3 functions as a bona fide eraser for H3K9la and H4K16la; structural analyses reveal that SIRT3 possesses a pre‐configured hydrophobic pocket that specifically accommodates the hydrocarbon moiety of lactyl‐lysine [79]. Based on these mechanistic insights, pharmacological activation of SIRTs—using agonists such as SRT2104, the SIRT3‐upregulating compound Honokiol [82], and the SIRT6 activator MDL‐800 [83]—has emerged as a potential strategy to suppress the CSC phenotype by restoring the epigenetic balance.

However, the therapeutic exploitation of delactylases is hindered by profound risks of epigenetic perturbation and functional contradictions. A primary concern is the pleiotropic nature of these enzymes. Because SIRTs and HDACs also function as broad‐spectrum deacetylases essential for DNA repair and metabolic homeostasis, their pharmacological activation may trigger non‐specific global epigenetic silencing—the indiscriminate removal of essential histone marks required for normal cellular function, potentially leading to severe systemic metabolic toxicity.

Furthermore, the diversity of the SIRT family introduces a “therapeutic paradox”. While activating SIRT1, 3, or 6 is generally associated with stemness inhibition, SIRT4 has been found to promote stemness in PDAC (primarily through its deacetylase activity) [35]. Consequently, the use of non‐selective pan‐SIRT activators may result in counterproductive effects by simultaneously stimulating pro‐stemness factors like SIRT4, thereby complicating drug design and increasing the risk of therapy failure.

Finally, the dual identity of HDACs as both deacetylases and delactylases creates a critical therapeutic dilemma. While HDAC inhibitors (HDACi) suppress tumors by increasing histone acetylation [84], they simultaneously block the removal of Kla marks. The resulting hyper‐lactylation risks paradoxically reinforcing CSC stemness, thereby driving therapy resistance.

#### 4.1.3 Targeting Readers: The Druggability Gap

The functional consequences of lactylation are decoded by “readers”—specialized proteins that recognize and bind to Kla marks to orchestrate downstream chromatin remodeling and gene transcription. To date, only three proteins have been conclusively identified as lactylation readers: Brg1 (SMARCA4), DPF2, and TRIM33.

Brg1, the core catalytic subunit of the SWI/SNF chromatin‐remodeling complex, specifically recognizes H3K18la via its bromodomain [22]. Given Brg1's established role in maintaining cancer stemness [85, 86], this interaction likely serves as an important mechanism driving the CSC phenotype. Furthermore, structural analyses of DPF2 and TRIM33 have elucidated the specific structural basis for Kla selectivity. Zhai et al. [87] demonstrated that the D274 residue within the DPF2 PHD1 domain specifically binds H3K14la using a mechanism distinct from acetyl‐lysine recognition. Similarly, a specific glutamate residue (E981) within the TRIM33 bromodomain pocket dictates selective Kla binding [88].

Despite these structural insights, clinically viable inhibitors targeting these specific reader domains remain undeveloped. Because reader proteins typically recognize epigenetic marks via relatively shallow protein‐protein interaction (PPI) surfaces rather than deep catalytic pockets, designing highly selective small‐molecule inhibitors is inherently challenging [89]. To effectively bridge this druggability gap, future interventions must explore innovative modalities, such as PROTAC‐mediated degradation of these non‐catalytic scaffolds, offering a powerful strategy to silence lactylation‐driven stemness programs.

### 4.2 Targeting Indirect Modulators of the Lactylation Network

Beyond the core “writers”, “readers”, and “erasers”, a diverse array of indirect regulators orchestrate lactylation levels and functions by modulating substrate availability, guiding complex assembly, or enhancing enzymatic activity. Targeting these “bridges” that integrate metabolic signals with the epigenetic landscape offers a promising strategy for reversing cancer stemness.

#### 4.2.1 Intercepting Substrate Supply: Nuclear Metabolic Enzymes

The localized supply of lactyl‐CoA is a critical limiting factor for lactylation efficiency. Beyond classical glycolysis, specific metabolic enzymes generate lactyl‐CoA directly within the nucleus. For instance, GTP‐specific succinyl‐CoA synthetase (GTPSCS) translocates to the nucleus to form a complex with p300, locally converting lactate to lactyl‐CoA and driving radioresistance in glioblastoma [90].

Significant progress has centered on Acyl‐CoA Synthetase 2 (ACSS2). In glioblastoma, EGFR activation triggers ERK‐mediated phosphorylation of ACSS2 at Ser267, promoting its interaction with Importin α5 and subsequent nuclear translocation. Once in the nucleus, phosphorylated ACSS2 directly converts lactate to lactyl‐CoA and, crucially, couples with KAT2A to form a functional complex. This complex catalyzes site‐specific H3K18/14 lactylation, which activates the Wnt/β‐catenin and NF‐κB pathways to drive tumor growth and immune evasion [64]. These findings underscore that metabolic enzymes function not merely as metabolic effectors but as core hubs integrating extracellular signaling with epigenetic regulation.

Consequently, intercepting this nuclear substrate supply has emerged as an attractive therapeutic strategy. Nevertheless, translating this concept into the clinic is hindered by three obstacles. First, the physiological essentiality of metabolic enzymes poses severe toxicity risks. Enzymes such as ACSS2 and GTPSCS serve fundamental roles in normal cellular metabolism [90, 91]. Long‐term or systemic pharmacological inhibition may disrupt global metabolic homeostasis, and it remains unclear whether a sufficiently wide therapeutic window exists to spare normal tissues. Second, target specificity remains a major concern. While ACSS2 inhibitors (e.g., VY‐3‐135) have shown activity in breast cancer models, it is uncertain whether existing acetate‐competitive inhibitors can effectively block lactylation‐mediated functions, given the potential structural differences in the binding pockets for acetate versus lactate [92]. Third, blocking peptides designed to intercept the ACSS2‐KAT2A interaction exhibit high selectivity and have been shown to enhance PD‐1 immunotherapy [64]; however, their in vivo stability and efficient delivery across the blood‐brain barrier (BBB) remain daunting pharmacological challenges.

#### 4.2.2 Disrupting Complex Assembly: LncRNAs and Scaffold Proteins

Long non‐coding RNAs (lncRNAs) frequently serve as spatial navigators, guiding the precise assembly of lactylation‐related complexes at specific chromatin loci. For example, in colorectal cancer, lncRNA STEAP3‐AS1 facilitates the recruitment of p300 by Brg1, inducing H3K18la enrichment at pro‐metastatic gene promoters [74]. In pancreatic cancer, lncRNA FLG‐AS1 guides the CTCF/HNRNPU complex to recruit p300, thereby elevating local lactylation [59]. Furthermore, the heterochromatin protein CBX3 functions as a molecular adapter that recruits p300, refining its substrate specificity toward lactyl‐CoA and thereby driving the Sox2 transcriptional program essential for maintaining glioblastoma stem cell traits [93].

Given the scaffolding roles of these macromolecules, several pharmacological strategies have been developed to disrupt complex assembly. For instance, Curaxin has been identified via molecular docking as a small‐molecule inhibitor of CTCF, which forms hydrogen bonds with residues R399, H340, Y343, and K344 within its DBR structural groove. In vitro and in vivo experiments have confirmed that Curaxin treatment significantly reduces CSF1 expression, suppresses tumor growth, and enhances sensitivity to gemcitabine in PDAC models [59]. Furthermore, while direct inhibition of CBX3 is hindered by its high structural similarity to other CBX family members, its pathway can be targeted indirectly. Given that CBX3 expression is regulated by BRD4 [94], the use of BRD4 inhibitors that are already in clinical investigation—such as ABBV‐075 (Molibresib), RO6870810, or AZD5153—may represent a more feasible strategy to disrupt CBX3‐mediated stemness and therapy resistance, although the specific efficacy of this approach in this context warrants further validation.

In summary, targeting indirect regulators offers a promising therapeutic avenue. However, this strategy is currently constrained by the inherent difficulties of drugging transcription factor interfaces, overcoming the delivery barriers of blocking peptides, and distinguishing closely related acyl‐CoA substrate pockets. Future therapeutic breakthroughs will therefore depend on developing high‐resolution agents capable of specifically disrupting the lactylation‐stemness axis without collapsing basal metabolic homeostasis.

### 4.3 Natural Products as Putative Modulators of Lactylation

Natural products have long held a pivotal position in anticancer drug discovery owing to their structural diversity and relatively favorable toxicity profiles. Recent studies provide preliminary evidence that certain natural compounds can suppress lactylation levels in tumor cells, offering an alternative dimension for reversing cancer stemness. For instance, the triterpenoid Demethylzeylasteral (DML), extracted from *Tripterygium wilfordii*, reduces lactate levels in liver CSCs, thereby inhibiting H3K9la and H3K56la modifications to trigger apoptosis and impair migratory capacity [95]. Similarly, in pancreatic cancer models, DML downregulates LDHA expression, indirectly lowering H3K18la levels [96]. Furthermore, Royal jelly acid (RJA) exerts antitumor effects by inhibiting glycolysis to downregulate H3K9la and H3K14la modifications in hepatocellular carcinoma [97]. Beyond isolated compounds, the traditional botanical formulation Ge Gen Qin Lian Decoction (GQD) alleviates ulcerative colitis progression by inhibiting LDH activity, thereby reducing global lactylation and specifically depleting marks at H3K18, H3K23, H4K8, and H4K12 [98]. Additionally, evodiamine [99] and tanshinone I [100] have been reported to decrease H3K18la levels, although their precise molecular targets remain uncharacterized.

Despite these encouraging observations, the current evidence base remains largely phenomenological. A primary confounding factor is that these anti‐lactylation effects are predominantly secondary consequences of upstream metabolic inhibition (e.g., restricted glycolysis or LDHA suppression) rather than targeted epigenetic modulation. Consequently, it is challenging to distinguish specific regulatory interventions from global shifts in metabolic flux. Furthermore, the inherent complexity of multi‐component herbal formulas introduces formidable obstacles in isolating the exact bioactive constituents. Most critically, there is a distinct lack of evidence demonstrating direct physical interactions between these natural products and the core lactylation machinery (writers, erasers, or readers). Therefore, to advance this emerging therapeutic landscape, the field must transition from phenomenological reporting to rigorous mechanistic validation. Identifying direct molecular targets and elucidating their precise structural binding modes with lactylation‐regulatory proteins are prerequisite steps to fully harness the translational potential of natural products.

## 5 Conclusions

Tumor stemness, the primary driver of cancer relapse, metastasis, and therapy resistance, is fueled by a sophisticated metabolic‐epigenetic regulatory axis. Rather than acting as a mere byproduct of lactate accumulation, lactylation functions as a critical epigenetic hub that shapes the CSC landscape across four functional dimensions: (i) regulation of core self‐renewal networks, (ii) modulation of stemness‐associated signaling pathways, (iii) orchestration of stress responses and microenvironmental adaptation, and (iv) induction of lineage plasticity and phenotypic reprogramming. Substantial evidence now underscores that lactylation is orchestrated by a dynamic network of “writers”, “readers”, “erasers”, and auxiliary regulators, with specific factors like p300, HDAC1, SIRT1, and CBX3 being experimentally validated as direct participants in cancer stemness regulation. This positions the lactylation network as an upstream regulatory node that critically influences tumor cell plasticity and drug resistance.

Building upon these mechanistic insights, therapeutic modulation of the lactylation network represents a promising anticancer frontier, though the current pharmacological toolbox is constrained by distinct, class‐specific hurdles. For instance, while broad‐spectrum agents—such as p300/CBP inhibitors and SIRT activators—have demonstrated preclinical efficacy in dampening lactylation and suppressing tumor growth, their clinical application is severely limited by risks of systemic toxicity and global epigenetic perturbation. Concurrently, alternative modulatory approaches are being explored, including metabolite‐competitive inhibitors (e.g., β‐alanine targeting AARS1) and bioactive natural products (e.g., DML and GQD). However, these strategies face unique limitations: the clinical utility of β‐alanine is restricted by the essential role of AARS1 in normal protein translation, whereas validating the precise mechanistic targets of complex natural products remains a formidable challenge. To achieve greater selectivity, the field is advancing toward targeted interventions, exemplified by ACSS2‐KAT2A blocking peptides and structure‐guided drug designs tailored to specific reader pockets (such as DPF2‐D274 or TRIM33‐E981). Nevertheless, the development of these highly selective agents is significantly hindered by inherent protein druggability gaps and in vivo delivery barriers.

Ultimately, advancing this field from preliminary pharmacological exploration to clinical reality will require integrating structural biology, advanced drug delivery platforms, and context‐specific combinatorial regimens to safely and effectively disrupt the metabolic‐epigenetic circuitry of CSCs.

## 6 Challenges and Future Directions

**FIGURE 3:**
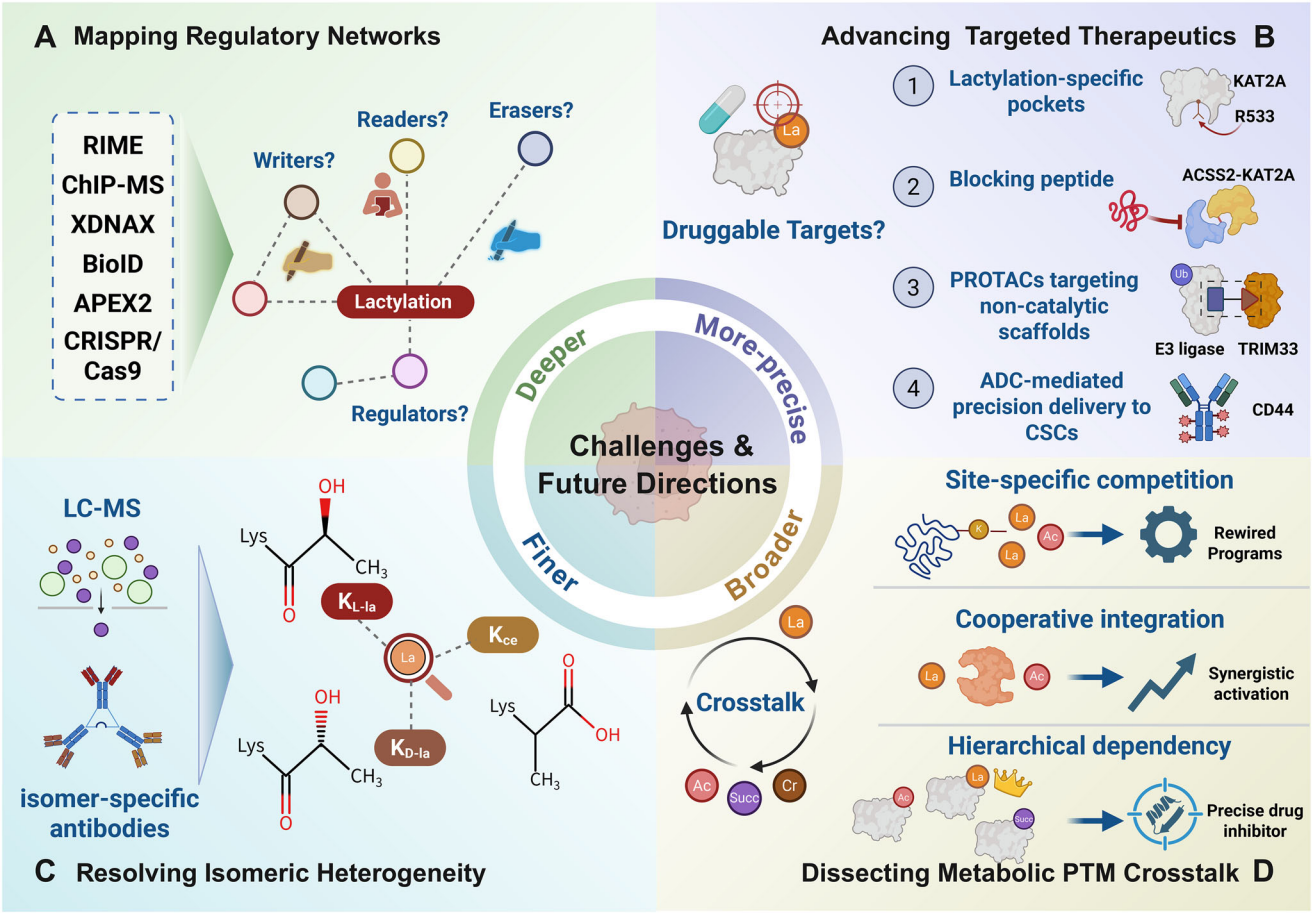
Challenges and strategic roadmap for future research on lactylation in cancer stemness. This figure summarizes the core challenges that must be addressed as lactylation research transitions from initial discovery to mechanistic exploration and clinical translation. (A) **Mapping regulatory networks**: Systematically identifying lactylation “writers,” “readers,” and “erasers” by integrating multidimensional tools such as RIME, ChIP‐MS, XDNAX, BioID, APEX2, and CRISPR/Cas9 screens. (B) **Advancing targeted therapeutics**: Moving beyond global inhibition to target specific druggable sites within the lactylation network including lactylation‐specific pocket inhibitors, blocking peptides, PROTACs targeting non‐catalytic scaffolds, and ADC‐mediated precision delivery to CSCs. (C) **Resolving isomeric heterogeneity**: Utilizing isomer‐specific antibodies and high‐resolution LC‐MS to precisely distinguish lactylation stereoisomers (K_L‐la_ and K_D‐la_) and carboxyethylation (K_ce_), thereby clarifying their distinct biological functions. (D) **Dissecting metabolic PTM crosstalk**: Unraveling the complex interactions between lactylation and other metabolic post‐translational modifications (e.g., acetylation, succinylation, crotonylation) across three key dimensions: site‐specific competition, cooperative integration, hierarchical dependency.

The field of lactylation research is transitioning from an era of initial discovery to a phase of intensified mechanistic exploration and the systematic identification of the regulatory landscape. To advance from phenomenological association to definitive causation and clinical translation, several pivotal challenges need to be addressed (Figure 3).

### 6.1 Mapping Comprehensive Regulatory Networks

The systematic delineation of the lactylation regulatory landscape represents a significant hurdle. Specifically, identifying a complete repertoire of ‘writers,’ ‘readers,’ and ‘erasers’ is complicated by the dynamic and often elusive nature of metabolic‐epigenetic signaling. While traditional immunoprecipitation coupled with mass spectrometry (IP‐MS) remains a cornerstone for protein discovery, it often struggles to capture the transient or low‐affinity interactions that characterize these signaling events. To overcome this bottleneck, future studies should strategically implement emerging proteomic technologies.

For precise identification within the chromatin environment, technologies such as RIME (Rapid Immunoprecipitation Mass Spectrometry of Endogenous Proteins) [101] or ChIP‐MS can be utilized to identify protein complexes at specific genomic loci in their endogenous state. Building on this, a recently developed “zero‐distance” photo‐crosslinking approach, termed XDNAX, offers a novel perspective. By utilizing an efficient UVED system for irradiation and metabolic DNA labeling with 4‐thiothymidine, this methodology enables the quantitative analysis of the DNA interactome in living cells at single‐amino‐acid resolution. This allows researchers to distinguish between proteins that are merely proximal to lactylation sites and those that physically engage the genome to drive transcription [102].

Furthermore, to capture unstable or transient regulators frequently lost during conventional washing steps, proximity‐labeling technologies such as BioID or APEX2 can be introduced. By labeling proteins in the vicinity of target enzymes in living cells, these tools facilitate the mapping of a more comprehensive spatial interactome. Finally, high‐throughput CRISPR/Cas9 functional screens will be essential for validating core regulatory nodes, enabling researchers to prioritize candidate genes whose depletion results in a significant lactylation‐inhibitory phenotype. Through the integrated application of these diverse technological approaches, the field will transition from descriptive observation toward a high‐resolution, systemic understanding of the lactylation machinery.

### 6.2 Advancing Precise Targeted Therapeutics

A critical translational challenge lies in distinguishing between biologically interesting targets and pharmacologically druggable targets. Many core regulators identified to date, such as p300, function as pleiotropic nodes essential for global epigenetic homeostasis. Pan‐inhibitors of these enzymes often encounter severe translational bottlenecks due to systemic toxicity and a lack of selectivity between acetylation and lactylation. To bridge this gap, future research must prioritize molecular specificity through structure‐guided designs targeting lactylation‐specific pockets. For instance, exploiting the R533 residue in KAT2A offers a structural rationale to selectively inhibit lactyl‐CoA utilization without interfering with basal acetylation [64].

Another promising strategy involves moving from global enzymatic inhibition toward targeting context‐specific protein‐protein interactions. Instead of suppressing an enzyme's entire catalytic activity, the development of blocking peptides, such as those disrupting the ACSS2‐KAT2A complex, offers a way to precisely interfere with the metabolic‐epigenetic machinery in CSCs [64]. Furthermore, for natural products like DML and GQD, identifying direct molecular targets remains essential to transition from phenomenological observations to definitive mechanistic characterization and validated pharmacological leads.

Beyond traditional small‐molecule inhibitors, exploring novel therapeutic modalities provides strategic advantages in targeting the unique vulnerabilities of CSC biology. Proteolysis‐targeting chimeras (PROTACs) offer a powerful platform for eliminating lactylation‐modifying proteins (e.g., TRIM33) that possess critical non‐catalytic scaffold functions. Concurrently, antibody‐drug conjugates (ADCs) could facilitate the precision delivery of lactylation‐modulating payloads specifically to cells expressing CSC surface markers, such as CD44. This transition from simple functional inhibition to targeted degradation and precision delivery is foundational for developing high‐potency, low‐toxicity interventions.

### 6.3 Resolving Isomeric Heterogeneity

Achieving superior structural precision is essential for unraveling the intricate roles of lactate‐derived modifications in cancer stemness. Because lactylation stereoisomers (K_L‐la_ and K_D‐la_) and carboxyethylation (K_ce_) often originate from distinct glyoxalase‐related pathways, failing to distinguish among them conflates their divergent biological functions. Therefore, future research should incorporate isomer‐specific antibodies and high‐resolution liquid chromatography‐mass spectrometry to precisely map these distinct chemical signatures.

The necessity of distinguishing these isomers is demonstrated by the specific role of D‐lactate in certain malignancies. For instance, in esophageal squamous cell carcinoma (ESCC), D‐lactate metabolism is specifically tailored to support the CSC phenotype through a dedicated CDK7‐YAP‐LDHD signaling axis. This axis facilitates the efficient clearance of D‐lactate in CSCs, preventing toxic accumulation and maintaining the metabolic homeostasis required for high energy demands and self‐renewal [103]. Dissecting the unique functional niches occupied by these diverse isomers will be vital for exposing distinct metabolic vulnerabilities uniquely required by the CSC subpopulation.

### 6.4 Dissecting Broader Metabolic PTM Crosstalk

The central challenge in advancing CSC research is dissecting the intricate crosstalk between lactylation and other metabolic PTMs. Rather than viewing these marks in isolation, future investigations should focus on how their intersection orchestrates the CSC landscape across three key dimensions: site‐specific competition, cooperative integration, and hierarchical dependency.

At the level of direct competition, antagonistic occupancy at shared lysine residues functions as a critical biochemical switch. As exemplified by p53, the competition between lactylation and acetylation at identical sites can antagonize conventional tumor‐suppressive functions, fundamentally rewiring established regulatory programs [69]. Instead of competing, distinct PTMs can also coordinately integrate diverse metabolic signals to synergistically support stemness. For example, simultaneous modifications on distinct residues of the same histone tail can recruit multi‐domain “reader” complexes to collaboratively activate stemness‐related transcriptional networks [104, 105]. Finally, resolving the hierarchical priority among these intersecting modifications is a critical next step. Clarifying which PTM acts as the primary driver and which serves as a subsequent adaptation will help pinpoint the foundational regulatory nodes within the stemness network.

In conclusion, lactylation research is rapidly maturing into a phase demanding comprehensive network mapping, precision‐targeted therapeutics, stereochemical resolution, and a broader integrative vision. Successfully navigating these challenges will not only solidify our understanding of cancer stemness but also pave the way for next‐generation targeted therapies to definitively overcome clinical treatment resistance.

## Author Contributions

**Ting Li**, **Hongyu Gu**, and **Chang Liu**: Writing – original draft, Visualization. **Yuesheng Lv** and **Jia Yi**: Writing – review & editing. **Feifei Li** and **Liang Liang**: Investigation. **Xuanyi Wang**, **Yi Zhun Zhu**, and **Chuan Xu**: Writing – review & editing, Supervision, Funding acquisition. All authors have read and agreed to the published version of the manuscript.

## Funding

This work was supported by grants from the National Key Research and Development Program of China (2023YFC3402100, 2026YFE0208100, and 2025YFA1109200), the National Science and Technology Major Project for Prevention and Treatment of Cancer, Cardio‐Cerebrovascular, Respiratory and Metabolic Diseases (2025ZD0544401), the National Natural Science Foundation of China (82541044 and 82403664), the Sichuan Province Natural Science Foundation Key Project (2024NSFSC0057), and the Major Project of Chongqing Natural Science Foundation (CSTB2024TIAD‐KPX0029).

## Conflicts of Interest

The authors declare no conflicts of interest.

## Data Availability

The authors have nothing to report.
